# Suppurative Cleft Palates and Devices for the Same

**Published:** 1904-02

**Authors:** Henry J. Janlusz

**Affiliations:** New York City


### SUPPURATIVE CLEFT PALATES AND DEVICES FOR THE SAME.[1]

BY HENRY J. JANLUSZ, D.D.S., NEW YORK CITY.

Mr. Chairman and Gentlemen.—It gives me great pleasure to appear before you on this occasion, and I assure you that I most thoroughly appreciate the courtesy and honor conferred upon me by being invited to deliver an address before you. My anticipations were very great when I knew I was to attend this meeting, but the fruition far exceeds anything I could have imagined. It is always most pleasant to come in contact with those who are interested in my work, or those whom I may interest.

The subject I wish to discuss to-day is one that admits of a most comprehensive description, readily and easily understood, and while it is in the nature of an innovation, I am quite confident I can bring clearly before you my method of treating certain forms of cleft palate. My work in this direction, and I say it without any flattery to myself, fills what I consider a long-felt want. It is a decided digression from the regular line of dentistry, but one of which I have made a deep study and a specialty. In my work I have sought neither praise, honor, notoriety, nor distinction. I am free to confess I have been inoculated with a certain amount of self-conceit, and while I do not look upon myself in the light of a beatified good Samaritan, I do consider that I have brought joy and happiness into many a discouraged brother's life, and, my friends, you can do the same. I am sure there is not one present who would not be willing to put aside all sensitive feelings if he could be assured that by so doing he would better the condition of suffering humanity.

But will you pardon this digression? I can only plead in extenuation of the fault that I feel so enthused with my work I am always most anxious to interest others, and for this reason it is often difficult to me to refrain from being too verbose.

I have made a specialty for some considerable time of treating cases of cleft palate where they were the result of either hereditary or acquired syphilis, a phase of the work about which little has been

---

[1] **Read before the Massachusetts Dental Society, June 3 and 4, 1903.**

written and almost as little done for the people upon whom this dread disease has been inflicted. I am free to confess that cases of this nature are far from inviting to handle; in fact, unless the greatest care is exercised the gravest results will occur. But there are other as dangerous patients with whom we come in almost daily contact in our profession, and only cautious care and watchfulness is necessary to ward off any evil results. You will readily understand that tools used for clients of this class can not properly be used for others, even though there be no danger, but the idea would be most obnoxious. Implements must be more than carefully cleaned and left for a long time in glyco-thymoline. Never fail to have the hands most carefully protected with rubber gloves, and under no circumstance ever bring them in contact with the lips or eyes. Glyco-thymoline will minimize the possibility of infection, and very often in the course of my work I immerse my glove-covered hands in this preparation.

I have always found it best to become, to a certain extent, well acquainted with my patients, for such I consider them, for the simple reason they will understand your interest in them, will have every confidence in you, and more readily and earnestly follow your every direction to the most minute detail. As a general thing I have found people afflicted with this disease decidedly diffident about speaking of their affliction, but once their confidence is gained they have no hesitancy about going into every detail that may be required to insure perfect success in your work.

Having everything carefully prepared for taking your impression, the cavity should be carefully cleansed with glyco-thymoline and then smeared thoroughly with sweet oil. Into the cavity is then placed some wax which has been softened, in this way securing an exact impression. Carefully remove this and cool it. Replace it, and with a napkin wrung out of hot water smooth the under surface of the wax until it is flush or even with the natural palate. Insert some little wire hooks into the wax so that the loops hang down to be readily embraced by the impression material, which is now used to take an impression of the mouth in the usual manner. It is absolutely necessary that the cast become perfectly hard before removal, so that when taken from the mouth the wax plug comes with it. It is now plain to the meanest comprehension that you have an accurate impression of the mouth and cleft, and no difficulty should, of course, be experienced in making a model.

No other material but gold should be used for the plate, and before moulding the model the cleft should be filled with wax, so that the plate is swaged across it. I know that in some instances rubber has been substituted, but I would not recommend this for an instant, as in cases of this kind absolute cleanliness should be the key-note, and this cannot be gained if rubber is used, no matter what care and precaution may be exercised.

As is well known, there is a great deal of discharge of an exceedingly disagreeable nature from the cleft palate of a syphilitic patient, and in order to arrange for this, and to obviate the necessity of this mucus passing again into the system, I have arranged for an appliance in the nature of a small cup or receptacle for the retention of a tiny sponge or bit of gauze of the finest texture. It is necessary, for obvious reasons, that this appliance of which I speak be removable, and this is accomplished by having it fitted to the plate by means of a slide or screw.

In order to secure the appliance referred to you have simply to fill in the abnormal cavity of the cleft, which appears in the model, with wax, and then proceed to make a Babbitt's metal cast as you would an ordinary gold denture.

It is not my desire to be prolix or to weary my listeners, but I wish to call your attention to a few special cases which I have treated during the past two years, and which I trust may be of some interest to you.

Case I.—Mr. P. S., aged sixty-four, was inoculated many years ago. I met the gentleman for the first time about seven years ago. I show you the condition of the maxilla as it was then. Mr. P. S. engaged an advertising dentist to make him an obturator, which was nothing but a vulcanized rubber plate, with a considerable amount of soft rubber to fill out the cavity. You can imagine the suffering of this unfortunate man, the soft rubber destroying the maxilla in a most terrible manner.

I now show you the present condition of the maxilla. The plate I show you here is but a copy of the original, consequently it is not as fine as the plate which he is wearing. You will notice a hollow box soldered upon the plate proper, which holds the contrivance with a screw. The patient removes the sponge daily, changing it for a fresh one soaked in glyco-thymoline, which keeps the cavity clean and wholesome. Prior to my making this palate for the patient, he swallowed the mucus flowing from the cavity, a rather disagreeable state of affairs. But since wearing this appliance he tells me he feels like a new man.

Case II.—S. G., aged forty, American birth; father, American, perfectly healthy; mother, American, syphilitic. I could not receive any further information.

The cleft made its appearance for the first time about three years ago. Teeth intact, free from caries, perfectly sound. Originally I made a gold plate with my usual appliances. About eight months afterwards an ulcer made its appearance on the uvula. You all know the difficulty of retaining a dressing upon it. As you perceive, an extension soldered to the plate proper upon which a movable slide was soldered permits the patient to change the dressing according to the direction of the surgeon. After several weeks the ulcer healed, the slide was removed, and the plate is worn at present without it, giving the patient entire satisfaction and enabling him to speak plainly.

Case III.—L. W., aged fifty-one, sailor by profession, an illiterate man, from whom I could not elicit sufficient information regarding family history.

When twenty-one years of age he married a Malay woman whom he brought to this country. He claims that the parents of the woman were inoculated with the disease. In all my experience with difficult cases this was no doubt the most severe. The duplicate of the plate which I show you has two distinct appliances. One serves as a holder for the sponge, which the patient removes before retiring, the other is used during the night, medicated cotton being applied.

It is hardly possible to exaggerate my success in this particular case; but if there is one person in this world who appreciates my work and is thankful he made my acquaintance, it is this man.

Case IV.—A well-known dentist of New York City, some two years ago, sent me a man, English by birth, who had an acquired syphilitic cleft palate. As this man had but six hours time before his departure, I could not make him a plate according to my original idea. He wore a gold plate made in England, to which the old-fashioned Ash & Sons' tubular teeth were fastened. The little appliance which you perceive here is nothing but an ordinary neck-tie holder, as I had no time to make him a proper appliance, and accidentally had the neck-tie holder in my possession.

I wish to state that there was a constant flow of mucus. Imagine, gentlemen, a man swallowing this discharge constantly. My idea was not to permit the mucus to run down his throat. I simply took an ordinary vulcanized hollow rubber finger, which he placed into the catch, allowing the mucus to run into this rubber finger, which was replaced by a new one whenever necessary. You can imagine how happy the man was after having found relief. Several months later he visited me, accompanied by his daughter, a very pretty young woman, aged twenty-two, but who has never been in the possession of a set of teeth. I show you the copy of the plate I made her. The discharge in her case was not as severe as her father's.

Case V.—This is a case of unusual interest. N. M., male, aged forty-four. The case is similar to any other syphilitic cleft, with the difference that both sides of the maxilla were destroyed. The six teeth, two bicuspids and two molars on each side, were worn down to such an extent that it was necessary to crown them to lengthen the bite. This plate is like all others, simply a copy; the slides are removable, serving as the holders for the dressing or sponge. This patient wears the plate with absolute comfort and perfect satisfaction.

Case VI.—J. L., West Indies British, informs me that syphilis has been prevailing in his family for years past. This was an absolutely dry case, only at times a very small quantity of mucus making its appearance.

Case VII.—This is also a very interesting case, that of a lady, E. S., whose husband acquired syphilis while a young man. He committed suicide about four years ago, having lived a very miserable life. The poor victim, his wife, who, as she claims, was absolutely healthy, of American parentage, suffers for the vices of her late husband. I have made the woman as comfortable as possible, but I have little doubt that eventually she will die a miserable death. I have also made for this patient an artificial nose, but as she feels delicate about the matter, I am unable to give you a *fac-simile*, or rather a *prima facie* evidence, but I can assure you that the woman is as comfortable as possible under the circumstances.

Case VIII.—B. F., who has been a sufferer from syphilis for the past sixteen years. Though caries has troubled him, I could not prevail upon him to have the six anterior teeth removed, although they are not of much use to him. You will notice that I could place but four teeth on each side. On the plate proper I have a box made of pink rubber, in which an antiseptic is placed. This receptacle catches the discharge constantly. Whenever it is filled the patient uses a syringe, cleansing the receptacle by putting in another piece of cotton saturated with glyco-thymoline or a weak solution of carbolic acid. Naturally it is not a pleasant thing for a patient to wear a plate of this nature, and I have made an improvement on the second plate. Instead of making a rubber box, I simply cut an ordinary rubber ball in two portions, and fasten it to the plate proper, permitting the mucus to be drained into it. The rubber ball is replaced every second or third day. It makes the plate lighter and more pleasant to wear, but I dare say that at best it is but a palliative measure.

Case IX.—This represents a simple case of syphilitic cleft palate. I wish to say, gentlemen, that my purpose is not to show beautiful workmanship; I simply wish to convey my idea, because I have made it a practice to always make a duplicate of each case. The original of this copy is far lighter and much prettier in form, etc. The manner in which this was made is as follows: when you are packing a case of this nature, though I very seldom use rubber for cleft palate work, after you have the surface packed, put a piece of ordinary rolled cotton into the cavity, covering it with rubber, then proceed by pressing the same as you would in an ordinary denture. Examine the work, if you should suspect that it may vulcanize porous. I usually make the so-called plumpers when I work to fill out the cheeks. In this case you will perceive the slide on the back part of the plate proper, which holds the sponge or any dressing which you may care to use.

Fellow-workers, I thank you for your attention and the interest you have manifested. A stranger,—a foreigner,—I have much with which to contend in an endeavor to make myself thoroughly understood, for I have not only to overcome the difficulties of proper enunciation, but also the rules of prosody and syntax. If there is any one point on which any member wishes more explicit detail it will be my pleasure to give the same either in public or private. Again expressing appreciation of the courtesy accorded me, I will only add that I will feel more than repaid for my trite efforts if I have placed my subject in such a light that some fellow-practitioner may bring relief to at least one afflicted sufferer.

